# *MECP2* germline mosaicism plays an important part in the inheritance of Rett syndrome: a study of *MECP2* germline mosaicism in males

**DOI:** 10.1186/s12916-023-02846-2

**Published:** 2023-04-20

**Authors:** Yongxin Wen, Jiaping Wang, Qingping Zhang, Xiaoxu Yang, Liping Wei, Xinhua Bao

**Affiliations:** 1 Department of Pediatrics, Peking University First Hospital, Beijing, China; 2 Department of Pediatric Neurology, Guangdong Women and Children Hospital, Guangzhou, Guangdong Province China; 3 Center for Bioinformatics, State Key Laboratory of Protein and Plant Gene Research, School of Life Sciences, Peking University, Beijing, China

**Keywords:** *MECP2*, Mosaicism, Germline, De novo, Rett syndrome

## Abstract

**Background:**

Germline mosaicisms could be inherited to offspring, which considered as *“*de novo*”* in most cases. Paternal germline *MECP2* mosaicism has been reported in fathers of girls with Rett syndrome (RTT) previously. For further study, we focused on *MECP2* germline mosaicism in males, not only RTT fathers.

**Methods:**

Thirty-two fathers of RTT girls with *MECP2* pathogenic mutations and twenty-five healthy adult males without history and family history of RTT or other genetic disorders were recruited. Sperm samples were collected and ten *MECP2* hotspot mutations were detected by micro-droplet digital PCR (mDDPCR). And routine semen test was performed at the same time if the sample was sufficient. Additionally, blood samples were also detected for those with sperm *MECP2* mosaicisms.

**Results:**

Nine fathers with RTT daughters (28.1%, 9/32) were found to have *MECP2* mosaicism in their sperm samples, with the mutant allele fractions (MAFs) ranging from 0.05% to 7.55%. Only one father with *MECP2* c.806delG germline mosaicism (MAF 7.55%) was found to have mosaicism in the blood sample, with the MAF was 0.28%. In the group of healthy adult males, *MECP2* mosaicism was found in 7 sperm samples (28.0%, 7/25), with the MAFs ranging from 0.05% to 0.18%. None of the healthy adult males with *MECP2* germline mosaicisms were found with *MECP2* mosaicism in blood samples. There were no statistical differences in age, or the incidence of asthenospermia between fathers with RTT daughters and healthy adult males with *MECP2* germline mosaicisms. Additionally, there was no linear correlation between MAFs of *MECP2* mosaicisms and the age of males with germline *MECP2* mosaicisms.

**Conclusions:**

Germline *MECP2* mosaicism could be found not only in fathers with RTT daughters but also in healthy adult males without family history of RTT. As germline mosaic mutations may be passed on to offspring which commonly known as *“*de novo*”*, more attention should be paid to germline mosaicism, especially in families with a proband diagnosed with genetic disorders.

## Background

In current common practice, a mutation in a proband is considered as “de novo” if the mutant alleles were detected in the peripheral blood DNA of the proband but neither in that of the parents. Theoretically, de novo mutations (DNMs) are mutations that newly occurred within one generation, which took place during gametogenesis or postzygotically [1, 2]. But it is not the truth. Mosaicism in the parental germ cells has been demonstrated to be a significant source of "DNMs" in the offspring. However, the exact timing, pace of mutation, and other characteristics of DNMs are still unknown. For further study, we focus on Rett syndrome (RTT), a neurodevelopmental disorder mainly caused by *MECP2* variants, as a study model to investigate the origin of *MECP2* variants.

Methyl-CpG-binding protein 2 (MeCP2), a multifunctional protein involved in transcriptional regulation and chromatin structure modulation, is essential for many cellular processes, especially neurodevelopment. The most relevant neurodevelopmental disorder associated with MeCP2 dysfunction is RTT, a disease characterized by loss of acquired purposeful hand skills, loss of acquired language skills, gait abnormalities, and stereotypic hand movements. RTT is a neurodevelopmental disorder affecting females almost exclusively, and it is one of the most common causes of intellectual disability in females, with an incidence of approximately 1/15 000 ~ 1/10 000 in female newborns.

According to previous studies, 94–96% of *MECP2* variants in sporadic cases of RTT were of paternal origin [3–5]. Paternal germline *MECP2* mosaicism has been reported in several studies [6]. However, all those studies were focused on fathers with RTT daughters, and the rate of *MECP2* mosaicism in adult males without RTT family history remained unknown. Whether the germline *MECP2* mosaicism aggregates in the fathers with RTT daughters or is a random event remains unknown. Germline mosaic mutations, which were commonly known as *“*de novo*”* in the past, may be passed on to offspring and cause disease onset. Therefore, further research on germline mosaic mutations will be of great significance to the understanding of the genetic mechanism and the prevention measures of disease. In the current study, not only *MECP2* germline mosaicisms of fathers with RTT daughters were studied, but ten *MECP2* hotspot germline mosaicisms were also detected in adult males without family history of RTT or other neurological disorders.

## Methods

### Subjects

Fathers with RTT daughters and healthy adult males without RTT family history were recruited in this study.

The inclusion criteria for fathers with RTT daughters were as followings: (1) aged from 20 to 45 years old; (2) with a daughter of RTT carrying one of the ten *MECP2* hotspot mutations (including c.316C > T, c.397C > T, c.455C > G, c.473C > T, c.502C > T, c.763C > T, c.806delG, c.808C > T, c.880C > T, c.916C > T); (3) the subject was in good health himself without a family history of other genetic disorders apart from RTT; (4) volunteered to participate in this study and signed the written informed consent.

The inclusion criteria for healthy adult males in this study were as followings: (1) aged from 20 to 45 years old; (2) the subject was in good health himself and without a history of genetic diseases; (3) without a family history of RTT or other inherited disorders; (4) volunteered to participate in this study and signed the written informed consent.

### DNA isolation

Sperm and blood samples of 32 fathers with RTT daughters and 25 healthy adult males were collected. The routine semen test was also performed at the same time if the sample size was sufficient. Sperm samples were purified with PureSperm 40/80 assay (Nidacon, Sweden). Genomic DNA from sperm was extracted using the phenol–chloroform extraction method, and genomic DNA from peripheral blood was extracted using a salting-out procedure as used in our previous study [6].

### Micro-droplet digital PCR (mDDPCR)

mDDPCR with single-molecule resolution was used to accurately measure mutant allele fractions (MAFs). The *MECP2* mutations carried by their offspring were detected for those with RTT daughters, and ten *MECP2* hotspot mutations were detected for the healthy adult males without family history of RTT or other genetic disorders. Additionally, those with sperm *MECP2* mosaicism were further tested the same mutation site in blood samples. To avoid potential contamination of low-fraction mutant alleles, DNA from different tissue types was sheared separately. Ultraviolet treatment was carried out after shearing DNA from each sample. mDDPCR analysis was carried out for the absolute quantification of MAFs. TaqMan genotyping assays targeting *MECP2* mutations were designed. The mutant allele was labeled with VIC fluorophore, whereas the wildtype allele was labeled with FAM fluorophore. (P/N: 4,331,349, Applied Biosystems, IDs provided in Table 1). Genotyping quantitative PCR (qPCR) was first performed on a StepOne Plus real-time system (Applied Biosystems by ThermoFisher) to test the performance of the assays. The validated genotyping system was subjected to the downstream digital PCR reactions. Droplet emulsions were generated from a Raindrop Source droplet generator. PCR amplification was carried out with a controlled temperature ramp of 0.5℃/s. Fluorescent droplets were detected on a RainDrop™ Sense droplet detector.

**Table 1:** *MECP2* mutations targeted by TaqMan assays

Nucleotide change	Amino Acid change	SNP ID	TaqMan assay ID
c.316C > T	p.(R106W)	rs28934907	AHRSQ7K
c.397C > T	p.(R133C)	rs28934904	AHMSYIO
c.455C > G	p.(P152R)	rs61748404	AHN1WOW
c.473C > T	p.(T158M)	rs28934906	AHCTC1V
c.502C > T	p.(R168*)	rs61748421	AHD2B7P
c.880C > T	p.(R294*)	rs61751362	AHABGPF
c.916C > T	p.(R306C)	rs28935468	AHBKEVN
c.763C > T	p.(R255*)	rs61749721	C_27532119_10
c.806delG	p.(G269Afs*20)	rs61750241	AHPAUU4
c.808C > T	p.(R270*)	rs61750240	AHQJS1C

Reference cDNA: NM_004992.3

Reference amino acid sequence: NP_004983.1

RainDrop Analyst V3 software was used for calculating MAFs with a binomial distribution. For each *MECP2* mutation, the signals of heterozygous patients were used for signal compensation because they had strong signals in both channels representing wild-type and mutant alleles. The compensation procedure was performed following the manufacturer's user guide (RainDance Technologies). The MAFs were calculated as the ratio between the number of the mutant targets and the sum of the numbers of the mutant and wild-type targets. The detection limit of mDDPCR was 10^–4^ alternative allele/total genomic copies. In addition to ensure the accuracy and reliability of the experimental results, those who simultaneously met the following conditions were regarded as positive for subsequent analysis: (1) MAF ≥ 0.01%; (2) the number of mutant droplets during amplification ≥ 10; (3) the total copies of the genome > 10,000.

### Statistical analysis

IBM SPSS 22.0 software was used for all statistical analysis. Data distributions were checked by the Shapiro–Wilk test. Where the data fit a normal distribution, we used the independent samples t-test; where it did not, we used a Mann–Whitney U test. Fisher’s exact test was used to compare the incidence of asthenospermia between the two groups. Spearman correlation analysis was used to assess the correlation between MAFs of *MECP2* mosaicism and age in those with *MECP2* germline mosaicism. A p-value < 0.05 was considered significant.

This study was approved by the Institutional Review Board at Peking University and the Ethics Committee of Peking University First Hospital under approval code IRB00001052-11,087.

## Results

### *MECP2* mosaicism in fathers with RTT daughters

**Fig. 1:**
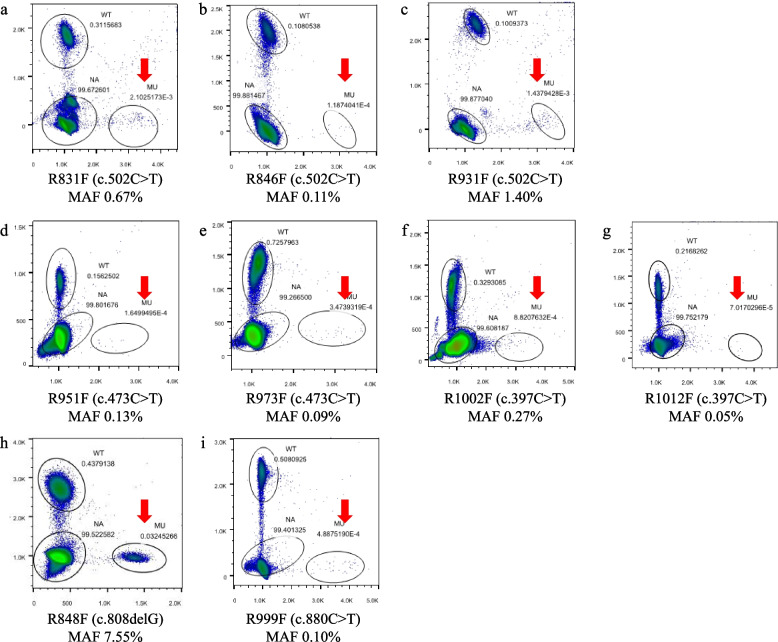
Germline *MECP2* mosaicism in fathers with RTT daughters. Mosaic variants are clearly demonstrated on the flow cytometry scatter plots of the mDDPCR results under the red arrow near “MU” at the bottom right corner. The 95% confidence intervals (CIs) for the mutant allele fraction (MAF = MU/[MU + WT]) were calculated under a binomial distribution. MU signals from mutant alleles, NA signals from droplets that did not contain target sequences and thus could not be amplified, WT signals from wild-type alleles

Totally, 32 fathers with RTT daughters were recruited, nine of them were identified with *MECP2* mosaicism in their sperm samples (28.1%, 9/32). The median MAFs was 0.13%, ranging from 0.05% to 7.55% (Fig. 1, results have been partially reported in our previous study [6]). The semen test was performed in 6 fathers with *MECP2* germline mosaicism (Table 2), of which two was asthenospermia (33.3%, 2/6). Among the *MECP2* mutation of the 32 daughters, 31 (96.9%, 31/32) were C to T transition. Out of the 9 germline *MECP2* mosaicisms, 8 (88.9%, 8/9) were C to T transition mutations.

**Table 2:** Clinical information of fathers with *MECP2* mosaicism

		**R831F**	**R846F**	**R848F**	**R931F**	**R951F**	**R973F**	**R999F**	**R1002F**	**R1012F**
**Age** **(years)**		26	29	35	30	33	30	36	37	35
**Nucleotide change**		c.502C > T	c.502C > T	c.806delG	c.502C > T	c.473C > T	c.473C > T	c.880C > T	c.397C > T	c.397C > T
**Amino acid change**		p.R168*	p.R168*	p.G269fs	p.R168*	p.T158M	p.T158M	p.R294*	p.R133C	p.R133C
**MAF in sperm**		0.67%	0.11%	7.55%	1.40%	0.13%	0.09%	0.10%	0.27%	0.05%
**MAF in blood**		0	0	0.28%	0	0	0	0	0	0
**Routine semen test**			NA					NA		NA
**Semen volume (mL)**		3	-	3	1	2	3.4	-	3.2	-
**Sperm density** **(million/mL)**		6.82	-	27.89	115.55	78	57	-	13.61	-
**Sperm motility**	a	10.34%	-	0	52.19%	12%	11%	-	12.38%	-
	b	3.45%	-	26.09%	18.08%	31%	24%	-	7.92%	-
	c	3.45%	-	30.44%	12.83%	10%	11%	-	9.9%	-
	d	82.76%	-	43.48%	16.91%	47%	54%	-	69.8%	-
(a + b)*(Density)*(volume) **(million/mL)**		2.05	-	21.84	81.2	67.08	67.83	-	8.84	-

Among the 32 fathers, 7 had daughters (21.9%, 7/32) with *MECP2* c.502C > T (p.R168*) mutation. The *MECP2* germline mosaic mutation of c.502C > T (p.R168*) was found in 3 (42.9%, 3/7) fathers (No. R831F, No. R846F and No. R931F), aged 26, 29 and 30 years old, respectively. The MAFs were 0.67%, 0.11%, and 1.40% (Fig. 1a-c). However, *MECP2* mosaicism was not found in their blood samples. The routine semen test showed normal sperm motility in No. R846F and No. R931F, but asthenospermia in No. R831F.

Three fathers had RTT daughters (9.4%, 3/32) with *MECP2* c.473C > T (p.T158M) mutation. Germline mosaicisms of this mutation were identified in 2 fathers’ sperm samples (66.7%, 2/3) (No. R951F and No. R973F), aged 33 and 30 years old, respectively. The MAFs were 0.13% and 0.09% (Fig. 1d-e), which were not found in their blood samples. The routine semen test of them was normal.

Three fathers had RTT daughters (9.4%, 3/32) with *MECP2* c.397C > T (p.R133C) mutation.

Two fathers (66.7%, 2/3) were found to have *MECP2* mosaicism in their sperm samples (No. R1002F and No. R1012F), aged 37 and 35 years old, respectively. The MAFs of *MECP2* mosaicism in their sperm samples were 0.27% and 0.05%, respectively (Fig. 1f-g). The routine semen test of R1002F showed asthenospermia, which was not tested for R1012F. Additionally, *MECP2* mosaicisms were not found in their blood samples.

Two fathers had daughters (6.3%, 2/32) with *MECP2* c.880C > T (p.R294*) mutation. The *MECP2* germline mosaicism of this mutation was found in one of them (50.0%, 1/2) (No. R999F), aged 36 years old. The MAF of *MECP2* germline mosaicism was 0.10% (Fig. 1i), which was not found in his blood sample. The routine semen test was not performed for No. R999F.

Only one father (R848F) aged 35-year-old had a daughter with *MECP2* c.806delG (p.G269fs) mutation. *MECP2* mosaicisms were found in both his sperm and blood samples, of which the MAFs were 7.55% (Fig. 1h) and 0.28%, respectively. The routine semen test showed roughly normal but close to asthenospermia.

In addition, 5 fathers had daughters with *MECP2* c.808C > T (p.R270*) mutation, 5 with c.916C > T (p.R306C), 4 with c.763C > T (p.R255*) and 2 c.316C > T (p.R106W) mutation, none of them were found with *MECP2* mosaicism in their sperm samples.

### *MECP2* mosaicism in healthy adult males without RTT family history

**Fig. 2:**
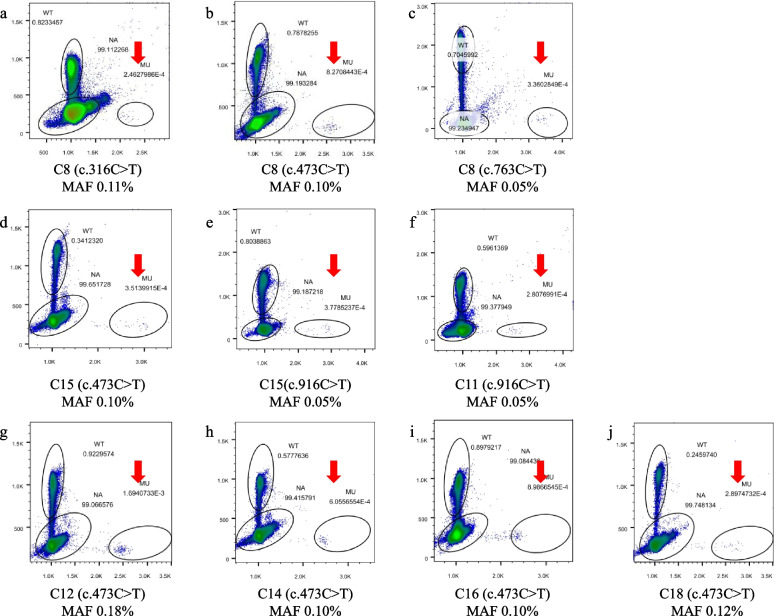
Germline *MECP2* mosaicism in adult males without RTT family history. Mosaic variants are clearly demonstrated on the flow cytometry scatter plots of the mDDPCR results under the red arrow near “MU” at the bottom right corner. The 95% confidence intervals (CIs) for the mutant allele fraction (MAF = MU/[MU + WT]) were calculated under a binomial distribution. MU signals from mutant alleles, NA signals from droplets that did not contain target sequences and thus could not be amplified, WT signals from wild-type alleles

A total of 25 healthy adult males were recruited in this study, aged from 24 to 39 years old. All of them were in good health, and without a family history of neurological or other genetic disorders. Ten *MECP2* hotspot mutations were analyzed in each sperm sample. Germline mosaic mutations were found in 7 healthy adult males (28.0%, 7/25). MAFs ranged from 0.05% to 0.18% (Fig. 2), and the median MAF was 0.10%. The semen test was performed in 7 healthy males with *MECP2* germline mosaicism (Table 3), of which one was asthenospermia (14.3%, 1/7). All the *MECP2* germline mosaicisms were C to T transition mutations. Totally, 6 cases (24.0%, 6/25) were found with the germline mosaic mutation of c.473C > T (p.T158M), 2 cases (8.0%, 2/25) with c.916C > T (p.R306C), 1 case (4.0%, 1/25) with c.763C > T (p.R255*), and 1 case (4.0%, 1/25) with c.316C > T (p.R106W). The mutation rate of ten *MECP2* hotspot mutation sites in sperm samples from adult males was 3.8*10^–4^ (the sum of MAFs of *MECP2* mosaicisms in adult males/25). The detailed information of those with germline *MECP2* mosaicisms was as follows.

**Table 3:** Clinical information of control males with *MECP2* mosaicism

		**C8**			**C11**	**C12**	**C14**	**C15**		**C16**	**C18**
**Age** **(years)**		28			36	33	32	29		38	29
**Nucleotide change**		c.316C > T	c.473C > T	c.763C > T	c.916C > T	c.473C > T	c.473C > T	c.473C > T	c.916C > T	c.473C > T	c.473C > T
**Amino acid change**		p.R106W	p.T158M	p.R255*	p.R306C	p.T158M	p.T158M	p.T158M	p.R306C	p.T158M	p.T158M
**MAF in sperm**		0.11%	0.10%	0.05%	0.05%	0.18%	0.10%	0.10%	0.05%	0.10%	0.12%
**MAF in blood**		0	0	0	0	0	0	0	0	0	0
**Routine semen test**											
**Semen volume (mL)**		2			1.3	2.3	3.1	2.6		2.4	1.1
**Sperm density** **(million/mL)**		75			67	47	34	68		75	13
**Sperm motility**	a	13%			8%	6%	8%	13%		10%	1%
	b	23%			18%	18%	14%	32%		18%	10%
	c	9%			10%	9%	11%	10%		11%	8%
	d	55%			64%	67%	67%	45%		61%	81%
(a + b)*(Density)*(volume) **(million/mL)**		54			22.65	25.94	23.19	79.56		50.4	0.20

No. 8 was a 28-year-old male, who neither smoked nor drank. Routine test of semen at 28 years old showed normal sperm motility. Mosaic *MECP2* mutation of c.316C > T (p.R106W), c.473C > T (p.T158M), and c.763C > T (p.R255*) were found in his sperm DNA, and the MAFs were 0.11%, 0.10%, and 0.05%, respectively (Fig. 2a-c). None of those *MECP2* mosaicisms was found in his blood DNA.

No. 15 was a 29-year-old male in good health, who drank a little sometimes and without offspring diagnosed with RTT or other genetic disorders. Routine test of semen at 29 years old showed normal sperm motility. *MECP2* c.473C > T (p.T158M) and c.916C > T (p.R306C) mosaicism were found in his sperm DNA, with the MAFs of 0.10% and 0.05%, respectively (Fig. 2d-e). However, *MECP2* mosaicism was not found in his blood sample.

No. 11 was a 36-year-old male, who never smoked nor drank and had a 9-year-old daughter in good health. Routine test of semen at 36 years old showed normal sperm motility. *MECP2* c.916C > T (p.R306C) mosaicism was found in his sperm DNA, of which the MAF was 0.05% (Fig. 2f). *MECP2* mosaicism was not found in his blood sample.

The *MECP2* c.473C > T (p.T158M) mosaicism was found in sperm DNA of No. 12, No. 14, No. 16, and No. 18 (Fig. 2g-j), aged from 29 to 38 years old, who were all healthy adult males and had no neurological family history, The MAFs of c.473C > T of these cases ranged from 0.10% ~ 0.18%. Routine semen tests of No. 12, No. 14, and No. 16 showed normal, but asthenospermia of No. 18. Additionally, *MECP2* mosaicism was not found in their blood samples.

### Statistical analysis

Because of non-normal distribution, the Mann–Whitney U test was adopted to detect the difference of age between fathers with RTT daughters and healthy adult males with *MECP2* germline mosaicisms. There was no statistical difference in age (P = 0.304) and the incidence of asthenospermia (*P* = 0.559) between fathers with RTT daughters and healthy adult males. Additionally, there was no linear correlation between MAFs of *MECP2* mosaicisms and age in males with germline *MECP2* mosaicisms (*r* = 0.008, *P* = 0.974).

## Discussion

Previous studies had reported several familial cases with RTT caused by *MECP2* gene mutation, but the mutation was absent in genomic DNA from both parents’ blood samples [7–9]. Germline mosaicism was one of the most likely explanations. However, most of the *MECP2* mutations in sporadic cases originated in the paternal germline X chromosome and could only be transmitted to females, which was called “paternal bias”. Paternal bias, the mutation detected in patients was more likely to be found in the paternal chromosome, has been reported in several monogenetic disorders such as Apert syndrome (caused by *FGFR2* gene variants), Noonan syndrome (caused by *PTPN11* gene variants), and achondroplasia (caused by *FGFR3* gene variants) [10, 11]. Our previous study did find *MECP2* mosaicisms in RTT fathers’ sperm cells [6], which indicated that “DNMs” were probably inherited from paternal germline mosaicisms. In order to further study the origin of “DNMs”, our current study focused on the pattern of paternal germline mosaicisms not only in the fathers of RTT but also in healthy adult males.

In this study, 28.1% fathers with RTT daughters and 28.0% the healthy adult males without RTT family history were found carrying *MECP2* mosaicisms in their sperm samples. Interestingly, multiple *MECP2* mosaic mutations were found in the same sperm samples of two healthy males without RTT family history in this study. *MECP2* mosaic mutations, c.316C > T, c.473C > T, and c.763C > T were found in sperm samples of No. 8, c.473C > T and c.916C > T were found in No. 15. It seems that sperm *MECP2* mosaicism was a random phenomenon in males rather than a specific feature in fathers with RTT daughters.

Among fathers with RTT daughters, c.502C > T (p.R168*) was the most common *MECP2* mosaicism in their sperm samples, followed by c.473C > T (p.T158M) and c.397C > T (p.R133C), which was basically consistent with their mutation rate in RTT patients according to our previous study [12]. The *MECP2* mosaic mutations in RTT fathers may be influenced by the sample offset (their daughters’ *MECP2* mutations), so we recruited healthy adult males without RTT family history for further study. The most common *MECP2* mosaic mutation among 25 sperm samples from the normal males was c.473C > T (p.T158M), which was also the most common *MECP2* mutation reported in the RettBASE database [13]. However, only 4 different *MECP2* mosaic mutations were identified in the 25 sperm samples from normal adult males, including c.473C > T (p.T158M), c.916C > T (p.R306C), c.316C > T (p.R106W), and c.763C > T (p.R255*). The spectrum of *MECP2* mosaic mutations was different between fathers with RTT daughters and healthy adult males, of which the limited sample size may be one of the potential explanations, further researches need to be based upon a larger sample size. Additionally, some mutation sites may have mosaicism tendencies in germ cells, which could be inherited to offspring and become the hotspot mutations in some diseases.

In the current study, the germline mutation rate of the ten *MECP2* hotspots of healthy adult males without RTT family history was 3.8*10^–4^. However, several studies have reported that the average human SNV mutation rate was approximately 1*10^–8^ per base pair per generation [14–16], which was much lower than the germline *MECP2* mutation rates detected in the current study. However, those studies were mostly based on the whole genome sequencing data from healthy parent–offspring trios, and most of the prior studies about germline mutation rates were estimated by mutations that passed on to offspring based on blood samples, not germ cells like egg or sperm directly. Our current study used mDDPCR, which has the most accurate detection limit of mosaicism and is performed with single-molecule resolution, to investigate the germline *MECP2* mutation rates directly in sperm samples. Therefore, we have reason to believe that our results would be much closer to reality. Additionally, as some females with *MECP2* mutations may present as asymptomatic, results would be more convincing if the *MECP2* gene test was performed for daughters of those with germline *MECP2* mosaicism.

*MECP2* mutations accounted for about 95% of RTT patients, of which nearly 99% were de novo. In our previous study, ten *MECP2* hotspot mutations were accounted for about 65% of RTT patients with *MECP2* mutations [12]. As the incidence of RTT ranged from 1/15000 to 1/10000, the mutation rate of the ten *MECP2* hotspots in females was about 4.1*10^–5^ ~ 6.1*10^–5^ (95%*99%*65%*1/15000 ~ 95%*99%*65%*1/10000). In this study, the mutation rate of the ten *MECP2* hotspot in sperm samples from adult males without RTT family history was 3.8*10^–4^. It was a little higher than that in females, which may be explained by the following points: Firstly, the *MECP2* mutation rate in females was calculated based on patients with clinical symptoms, however, due to X chromosome inactivation, some females with *MECP2* mutations may present as asymptomatic [9, 17]. Secondly, the conception rate of mutant sperms may be lower than wild-type sperms. Hastings et.al suggested that some mutations may have positive selection in the germ line, but have negative selection when transmitted to the offspring [18], resulting in a lower mutation rate in the offspring. Therefore, we suspected that most of the *“*de novo*” MECP2* mutations originated from sperm mosaicisms.

Mutations could occur at any time, and germline mosaicisms could be inherited to offspring, which considered as *“*de novo*”* in most cases. Primordial germ cells are differentiated from somatic progenitors in the first three weeks after conception in humans, and hematopoietic progenitors separate later from mesoderm [19]. As a result, mosaicisms detected in sperm samples are less likely to be detected in other tissues, whereas those detected in blood samples were often detected in saliva. Based on the cells and the original time of mosaicism, sperm mosaicism could be divided into three types [20]: Type I (sperm) mosaicism occurs in terminally or near-terminally postmitotic spermatocytes and sperm cells. Type II (spermatogonial stem cell, SSC) mosaicism occurs in SSCs and often accumulates by age. And Type III (embryonic) mosaicism occurs during paternal embryogenesis, which further divided into IIIa and IIIb. Type IIIa is also associated with evidence of mosaicism in somatic tissues like blood or saliva, whereas type IIIb limits to sperm. Most of the *MECP2* mosaicisms in our study were only found in sperm samples, but not blood samples in adult males. And there was no linear correlation between age and MAFs of *MECP2* mosaicism in males with *MECP2* germline mosaicisms, which suggested that most of the sperm *MECP2* mosaic mutations were type I or type IIIb mosaicism other than type II. Although prior studies have reported that mosaic variants accumulate in paternal germline cells with age because of constant meiosis and “selfish spermatogonial selection” [21, 22], however, a recent study identified that sperm clonal mosaic mutations were likely embryonic in origin and stable over age [19]. In the current study, only one father was found with *MECP2* c.806delG (p.G269fs) mosaic mutation in his sperm and blood samples, as well as saliva sample as we previously reported. The *MECP2* mosaicism detected in multiple tissues could be classified as type IIIa mosaicism, which may arise in early embryogenesis in this father and spread throughout his body. Additionally, for this subject, the MAF in germ cells was higher (7.55%) than that in blood (0.28%), for which a possible explanation was that the primordial germ cells of males undergo methylation reprogramming twice during embryonic development but only once in other tissues [23], as a result, genomic DNA of sperm cells are more unstable than other tissues. And methylated cytosine could spontaneously deaminate to thymine. All the *MECP2* mosaic mutations that only found in sperm cells were C to T transition, which might be closely related to the high methylation in sperms and the spontaneous deamination of methylated CpG [24].

Those with germline *MECP2* mosaicism may also have an opportunity to have a child with RTT, and paternal germline *MECP2* mosaicism may be an important factor to assess the risk of disease onset of offspring. Identification of germline mosaic mutations would have great implications for patients and their families. Nearly 80% DNMs are paternal in origin [25], and mosaicisms in germ cells may explain 3% ~ 8% of DNMs risk in monogenic diseases [26–28]. If DNMs occurred exclusively in germ cells, the recurrence risk would be negligible [2]. But actually, novel mutations can and do occur at any stage of gametogenesis and development [29]. Therefore, sperm mosaicism plays an essential role in assessing DNMs risk in offspring. Detecting the mutation rates of DNMs in germ cells like sperms directly would better assess the risks of particular disorders.

Ten *MECP2* hotspot mutation sites were analyzed in each sperm sample of normal adult males. However, only one site carried by their affected daughters was analyzed in each RTT father’s sperm sample, which may lead to some experimental bias to a certain degree. There may be other *MECP2* mosaic mutations in sperm cells of fathers with RTT daughters, and mosaic mutations of other genes cannot be ruled out. If the samples are sufficient, *MECP2* mutation sites should be tested as many as possible, which is conducive to the comprehensive analysis of the distribution characteristics of *MECP2* mosaicism in sperm samples. Additionally, only one single sample was taken from each subject in this study, which may not fully represent the whole picture of the germline mosaicism in males. If sperm samples from each participant could be collected and detected at different times, the results may be more convincing. As the detection limit of mDDPCR was 10^–4^, germline *MECP2* mosaicism with lower MAFs could not be ruled out as well. Further researches are needed for a better understanding the germline mosaicisms.

## Conclusions

In conclusion, germline *MECP2* mosaicism could be found in not only fathers with RTT daughters but also healthy adult males without family history of RTT. The *MECP2* mosaicism mutation in the sperm of general males suggests that germline *MECP2* mosaic mutations may occur randomly in the general population. The quite high rate of germline mosaicism of males observed in the current study led us to reconsider the concept of *“*de novo*”* mutations. In the future, more attention should be paid to germline mosaicism, especially in families with a proband diagnosed with genetic disorders.

## Data Availability

The datasets used and analysed during the current study are available from the corresponding author on reasonable request.

## Abbreviations

DNMs: de novo Mutations
RTT: Rett syndrome
MeCP2: Methyl-CpG-binding protein 2
MAFs: Mutant allele fractions
mDDPCR: Micro-droplet digital PCR
